# Complete chloroplast genome of Ulleung Island endemic pokeweed, *Phytolacca insularis* (Phytolaccaceae), in Korea

**DOI:** 10.1080/23802359.2018.1535841

**Published:** 2018-11-21

**Authors:** Ji Young Yang, Woong Lee, Jae-Hong Pak, Seung-Chul Kim

**Affiliations:** a Department of Biology, Research Institute for Dok-do and Ulleung-do Island, School of Life Sciences, Kyungpook National University, Daegu, Republic of Korea;; b Department of Biological Sciences, Sungkyunkwan University, Suwon, Republic of Korea

**Keywords:** Chloroplast genome, *Phytolacca insularis*, Ulleung island, Phytolaccaceae, Caryophyllales

## Abstract

The first complete chloroplast genome sequences of Korean endemic pokeweed in Ulleung Island, *Phytolacca insularis*, were reported in this study. The *P. insularis* plastome was 156,419 bp long, with the large single copy (LSC) region of 86,106 bp, the small single copy (SSC) region of 18,335 bp, and two inverted repeat (IR) regions of 25,989 bp. The plastome contained 132 genes, including 84 protein-coding, eight ribosomal RNA, and 39 transfer RNA genes. The overall GC content was 36.8%. Phylogenetic analysis of 13 representative plastomes within the order Caryophyllales suggests that *P. insularis* is closely related to the species in family Aizoaceae.

The genus *Phytolacca* L. belongs to family Phytolaccaceae and consists of about 25 species of perennial herbs, shrubs and rarely trees. The genus shows nearly cosmopolitan distribution, mostly native to South America and a few species in Africa and Asia (Nowicke 1969; Shu 2003). *Phytolacca* is a pharmaceutically important genus owing to several active compounds in leaves and fruits for analgesic, anti-inflammatory, bactericidal, and fungicidal actions (Hernandez et al. 2013). In particular, *P. acinosa* Roxb. in East Asia is known as ‘Phytolaccae Radix’, one of the important oriental herbal medicines for diverse biological properties, such as antibacterial, anti-inflammatory, antidiuretic, antiviral, and anticancer (Zhang et al. 1990; Gao et al. 2009). Also, berries and young leaves were utilized as an adulterant of red wine and made into poke salad, respectively (Nowicke 1969). Of 25 species of *Phytolacca*, one native (*P*. *insularis* Nakai) and two introduced species (*P*. *acinosa* and *P*. *americana* L.) are known in Korea (Lee, 2004). *Phytolacca insularis* occurs exclusively on Ulleung Island in East Sea (Nakai 1918; Kim 2004). Taxonomic status as a distinct insular endemic and relationship to other congeneric species are highly controversial. For example, Hong (2007) suggested that *P*. *insularis* is closely related to *P*. *japonica* Makino based on vegetative and reproductive traits, while Chae et al. (2007) recognized *P*. *insularis* as a form of *P*. *acinosa* based on pollen and seed morphology and ITS sequences. Thus, it is yet to be determined the distinct taxonomic status of *P. insularis* and phylogenetic relationships within the genus. For the first time, we sequenced the complete plastome of *Phytolacca*, *P. insularis*, and assessed phylogenetic position within Caryophyllales.

Total DNA (Voucher specimen: N37°30′26.4″ E130°49′24.7″, Lee171010135, KNU) was isolated using the DNeasy plant Mini Kit (Quiagen, Carlsbad, CA) and sequenced by the Illumina HiSeq 4000 (Illumina Inc., San Diego, CA). A total of 3,664,7851 paired-end reads were obtained and assembled *de novo* with Velvet v. 1.2.10 using multiple *k*-mers (Zerbino and Birney 2008). The tRNAs were confirmed using tRNAsacn-SE (Lowe and Eddy 1997). The total plastome length of *P*. *insularis* (MH376309) was 156,419 bp, with large single copy (LSC; 86,106 bp), small single copy (SSC; 18,335 bp), and two inverted repeats (IRa and IRb; 25,989 bp each). The overall GC content was 36.8% (LSC, 34.7%; SSC, 30.4%; IRs, 42.7%) and the plastome contained 132 genes, including 84 protein-coding, eight rRNA, and 39 tRNA genes. A total of 19 genes were duplicated in the inverted repeat regions including eight tRNA, four rRNA, and six protein coding genes. The complete *ycf*1 gene was included in the IR at the SSC/IRa junction and the complete *inf*A gene was located in LSC. The partial *ycf*1 gene became a pesudogene and located at IRb/SSC junction.

To confirm the phylogenetic position of *P*. *insularis*, 13 representative species of Caryophyllales were aligned using MAFFT v.7 (Katoh and Standley 2013) and maximum likelihood (ML) analysis was conducted using IQ-TREE v.1.4.2 (Nguyen et al. 2015). The ML tree showed that *P*. *insularis* (Phytolaccaceae) is sister to Aizoaceae (Figure 1).

## Figures and Tables

**Figure 1.**
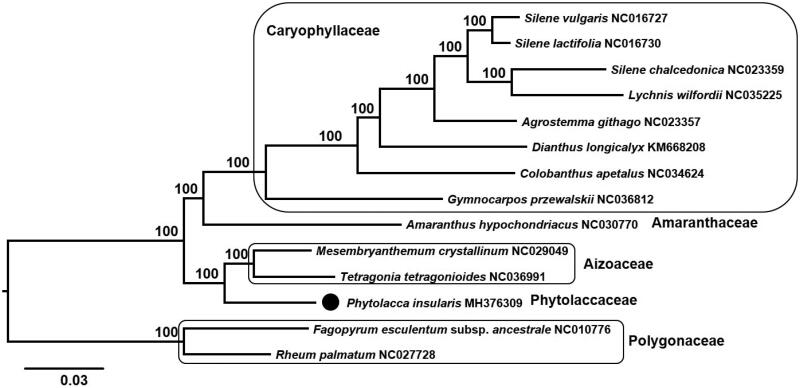
The maximum-likelihood (ML) tree based on the 13 representative chloroplast genomes of order Caryophyllales. The bootstrap value based on 1000 replicates is shown on each node.
